# mir-276a Is Required for Muscle Development in *Drosophila* and Regulates the FGF Receptor *Heartless* During the Migration of Nascent Myotubes in the Testis

**DOI:** 10.3390/cells14050368

**Published:** 2025-03-03

**Authors:** Mathieu Preußner, Maik Bischoff, Susanne Filiz Önel

**Affiliations:** 1 Department of Biology, Developmental Biology, Philipps University Marburg, Karl-von-Frisch Str. 8, 35037 Marburg, Germany; preussner@bio.uni-frankfurt.de (M.P.); maik.bischoff@unc.edu (M.B.); 2 Department of Developmental Biology of Vertebrates, Institute of Cell Biology and Neuroscience, Goethe University Frankfurt am Main, 60438 Frankfurt am Main, Germany; 3 Department of Biology, University of North Carolina at Chapel Hill, CB#3280, Chapel Hill, NC 27599-3280, USA; 4 Department of Biology, Molecular Embryology, Philipps University Marburg, Karl-von-Frisch Str. 8, 35037 Marburg, Germany

**Keywords:** myoblast fusion, testis muscle, larval body wall muscles, indirect flight muscles, IFM, DLM, microRNAs

## Abstract

MicroRNAs function as post-transcriptional regulators in gene expression and control a broad range of biological processes in metazoans. The formation of multinucleated muscles is essential for locomotion, growth, and muscle repair. microRNAs have also emerged as important regulators for muscle development and function. In order to identify new microRNAs required for muscle formation, we have performed a large microRNA overexpression screen. We screened for defects during embryonic and adult muscle formation. Here, we describe the identification of mir-276a as a regulator for muscle migration during testis formation. The mir-276a overexpression phenotype in testis muscles resembles the loss-of-function phenotype of *heartless*. A GFP sensor assay reveals that the 3′UTR of *heartless* is a target of mir-276a. Furthermore, we found that mir-276a is essential for the proper development of indirect flight muscles and describe a method for determining the number of nuclei for each of the six longitudinal muscle fibers (DLMs), which are part of the indirect flight muscles.

## 1. Introduction

The muscles of vertebrates and *Drosophila* are composed of large elongated multinucleated cells that arise through the fusion of mono-nucleated myoblasts. The determination of myoblasts and their ability to migrate, adhere, and rearrange their actin cytoskeleton, as well as undergo membrane merger, is essential for both muscle building and muscle repair. The somatic muscles of *Drosophila* arise from the mesoderm expressing high levels of the transcription factor Twist [1]. Within this domain, a myogenic cluster is defined by the expression of the transcription factor Lethal-of-Scute (L’Sc). Notch-mediated inhibition restricts L’Sc expression to one cell that becomes the progenitor cell [2]. The remaining cells of the myogenic cluster turn into fusion-competent myoblasts. The progenitor cell divides asymmetrically and gives rise either to two founder myoblasts or to a founder myoblast and one adult muscle precursor (AMP) cell [3,4]. In *Drosophila*, myoblast fusion occurs between founder cells and fusion-competent myoblasts [5,6]. Fusion-competent myoblasts are characterized by the expression of the Gli superfamily transcription factor Lame duck (Lmd) [7,8]. After the fusion process, the fusion-competent myoblasts are reprogrammed and express the characteristic transcription factors of the founder cell [9]. Thus, founder cells determine the identity of the muscle, e.g., shape, size, position, and attachments.

The AMP cells are considered muscle-committed transient stem cells that become activated during larval development and undergo extensive proliferation [10]. The adult structures of *Drosophila* derive from larval imaginal discs. The muscles of the male reproductive system are located at the genital disc. The genital disc originates from the embryonic tail segments A8/A9/A10 [11]. In contrast, the testes are of gonadal origin located at segment A5. During metamorphosis, the genital disc and the pupal testes grow toward each other. The myoblasts of the genital disc fuse to form the muscle sheaths that cover the male reproductive system [12]. The fusion of these myoblasts occurs at the region of the prospective seminal vesicles, a juvenile hormone-responsive tissue in adult males. Similar to embryonic myoblasts, founder cells and Lmd-expressing fusion-competent myoblasts have been identified in this myoblast population [12]. The fusion of testes myoblasts occurs at the anterior tip of the prospective seminal vesicles at 28 h after pupal formation (APF) [12,13]. The newly formed myotubes migrate collectively to populate and shape the entire testis [14,15,16].

Flight muscles are located at the thorax and can be distinguished into small direct flight muscles that control the angle of wing movement and indirect flight muscles that generate the power for flying [17]. Indirect flight muscles are divided into the dorsal longitudinal muscles (DLMs) and the dorsoventral muscles (DVMs). During metamorphosis, the muscles that have been built during embryonic stages undergo histolysis. However, the dorsal oblique muscles 1, 2, and 3 escape histolysis and serve as templates for the DLM muscles [17]. The fusion of myoblasts to these templates occurs 12–20 h APF. The fusion process initiates the splitting of the template muscles into six DLM muscles. A disturbance in template formation or in myoblast fusion negatively influences DLM muscle formation [17,18]. In contrast to DLMs, DVMs arise through de novo fusion.

The myoblasts that fuse to form the indirect flight muscles derive from the AMPs that undergo rapid proliferation during larval development and pupal metamorphosis. AMP proliferation is governed by signaling pathways involving Wingless, Notch, and FGF [19,20,21,22]. The AMP population migrates as a swarm from the imaginal disc but has not fully differentiated into myoblasts. The activation of Notch in some of these cells induces Twist expression, which suppresses the expression of fusion-relevant genes [23]. Only when the cells are in the vicinity of the template muscles do they start to express genes known to be active in fusion-competent myoblasts [23].

microRNAs are a family of small, noncoding, single-stranded RNA molecules that are approximately 22 nucleotides long. They serve as important regulators of gene expression. They guide the RNA-induced silencing complex (RISC) to a messenger RNA (mRNA) by binding to its 3′UTR [24]. This leads to the degradation of the mRNA and thus inhibits its translation. An aberrant expression of microRNAs is associated with many human diseases [25].

In this study, we investigate the function of the small microRNA mir-276a during muscle formation. We have expressed UAS-*mir-276a* in myoblasts using the GAL4 driver line of the *Drosophila* Myocyte enhancer factor-2 (D*Mef2*), a muscle differentiation gene [26,27]. We demonstrate that the overexpression of mir-276a causes defects during embryonic, testis, and indirect flight muscle development. To characterize myoblast fusion defects in DLMs, we present an approach on how to count the number of nuclei of DLMs. Based on the phenotypic similarities between the loss of FGF signaling and the overexpression of mir-276a during testis muscle migration, we gain evidence that the 3′UTR of *heartless* is a target of mir-276a.

## 2. Materials and Methods

### 2.1. Fly Stocks and Drosophila Genetics

Flies were grown on corneal agar at 25 degrees. The microRNA collection was obtained from FlyORF (University of Zurich, Switzerland). The following fly strains were used: D*Mef2*-GAL4 [28], UAS-*Dcr-2*, D*Mef2*-GAL4 (BL25756), UAS-*htl^RNAi^* (BL35024), UAS-*htl^DN^* (BL5366), UAS-*stumps^RNAi^* (v21317), and UAS-mCD8-GFP (BL32186). Flies carrying a BL number were obtained from Bloomington *Drosophila* Stock Center (Bloomington, IN, USA), and UAS-*stumps^RNAi^* was ordered from Vienna *Drosophila* RNAi Center (Vienna, Austria).

To generate UAS-*htl3′UTR*-eGFP transgenic lines, we amplified the 3′UTR from the *htl* cDNA LD32130 (obtained from the Berkeley Drosophila Genome Project/BDGP, CA, USA) and cloned it into the pUAST-attB-rfa-eGFP vector [29]. The following primers were used to amplify the *htl* 3′UTR: htl3UTR_f CACCACGAATCAGGATCCTTAGATGAGA and htl3UTR_rev GGGATTCGTGTACAACATTTAT.

The resulting construct was injected into *Drosophila* embryos of the ZH-86Fb line, carrying an attP site on chromosome arm 3R. Injected animals were crossed with white mutant flies, and the offspring were screened for *white*-positive animals. These animals were established by using double balancers.

### 2.2. Immunostaining and Confocal Microscopy

Pupae selection and genital disc dissection were carried out as described by Kuckwa et al. [11]. The following antibodies were used: anti-β3-Tubulin (1:10,000) [30], anti-Mef2 (1:500), and anti-Lmd (1:500), which were a gift from Hanh T. Nguyen. The following secondary antibodies were used: anti-guinea-pig Alexa Fluor^®^ 488 (1:500, Jackson ImmunoResearch Laboratories, Cambridge, UK) and anti-rabbit Fluor^®^ 589. The actin cytoskeleton was stained by using Alexa Fluor Phalloidin 568 (Thermo Fisher Scientific Inc., Passau, Germany), and nuclei were stained using DAPi (Thermo Fisher Scientific Inc., Passau, Germany). The testes and genital discs were imaged using a Zeiss AxioObserver Z.1 image microscope with the ApoTome function. Embryos and flight muscles were imaged using confocal microscopy using a Leica TCS SP8.

### 2.3. Paraffin Sections and Hematoxylin Staining

The preparation of paraffin-embedded flies for longitudinal sections was carried out as described by Kucherenko et al. [31]. Paraffin blocks were cut with 7–10 μm sections on a microtome.

For hematoxylin staining on adult flies, the adult flies were prepared out of the pupal case and fixed overnight in 4% paraformaldehyde in PBS. The fly was pinned at the head and the abdomen to remove the wings and the legs. Afterward, the thorax was horizontally cut into two pieces. The head and the abdomen were removed, and the thorax halves were stained for two minutes in hematoxylin, washed in PBS, and embedded in 80% glycerol.

### 2.4. Counting of Nuclei

Forty APF-old pupae raised at 25 °C were fixed on a double-sided tape to facilitate removal of the pupal case. After removal of the pupal case, pupae were transferred into PBS containing 0.3% Triton. A fine spring scissor was used to make a ventral incision beginning at the head, dividing the pupae into two halves as described by Weitkunat and Schnorrer [32]. Fat bodies and other tissues were carefully extracted with forceps and subsequently rinsed out using a pipette.

D*Mef2*-GAL4-driven eGFP expression in the IFM allowed us to identify the musculature. Subsequently, the samples were fixed in 4% paraformaldehyde in PBS for 20 min and washed three times for 10 min in PBS containing 0.3% Triton. IFMs were incubated for 30 min in DAPi (1:500, Thermo Fisher Scientific Inc., Passau, Germany) and Alexa Phalloidin 568 (1:500, Thermo Fisher Scientific Inc., Passau, Germany) and washed three times for 10 min in PBS containing 0.3% Triton. Afterward, the halves were mounted in Fluoromount-G.

The IFMs were imaged using confocal microscopy (Leica TCS SP8). The 405 nm laser intensity remained the same for all images. A z distance of 0.45 µm was set for the recording of the DLMs.

The Imaris 9.3 software was used to quantify the individual nuclei of the flight musculature. Using the “Surface module”, the individual DLM muscles were segmented by manually outlining each muscle across the different Z-planes. The created surface corresponds to one of the six DLM muscles. This generated surface is, therefore, masking the DAPi channel, ensuring that only nuclei within the defined surface are visible. The Spots module is subsequently applied to this region to count all nuclei automatically. The parameters for nuclear counting are set in a wizard and applied to all images. The “estimated XY diameter” is set to 3.50 µm in combination with background subtraction.

## 3. Results

### 3.1. Expression of mir-276a in Myoblasts Causes Defects in Larval Body Wall and Testis Muscles

**Figure 1:**
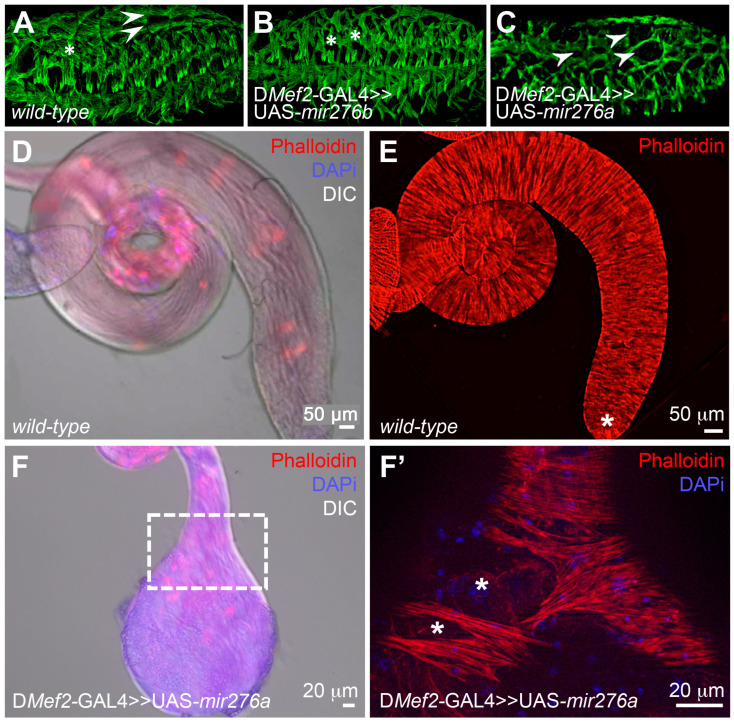
The expression of UAS-*mir276a* causes defects during muscle formation: (**A**–**C**) lateral view of staged 16 embryos stained with anti-β3-Tubulin to visualize the muscle pattern. 20× Objective (**A**) Wild-type muscle pattern of a homozygous D*Mef2*-GAL4 embryo. The asterisk marks the lateral transverse muscles. The arrowheads point to the dorsal muscles. (**B**) Embryo expressing UAS-*mir276b* with D*Mef2*-GAL4. Lateral muscles appear shorter in comparison to the lateral muscles of D*Mef2*-GAL4 embryos (asterisks). (**C**) Embryo expressing UAS-*mir276a* with D*Mef2*-GAL4. Large gaps between the dorsal muscles are seen (arrowheads). (**D**,**E**) Testis of wild-type adults showing an elongated coiled-like testis. Scale bar D 50 μm. (**E**) Muscles of a wild-type testis covering the whole testis, including the tip of the testis, asterisk. Scale bar 50 μm (**F**) Testis of a male expressing UAS-*mir276a* with D*Mef2*-GAL4. Scale bar 20 μm. (**F’**) Testis muscles of the boxed area in (**F**) are shown in a higher magnification. Holes in the musculature are marked with asterisks. Scale bar 20 μm.

To identify microRNAs (miRNAs) that are required during myoblast fusion, we screened the microRNA library of the inducible UAS-*miRNA* collection from FlyORF [33] and overexpressed these miRNAs using the muscle GAL4 driver lines D*Mef2*-GAL4, UAS-*Dcr-2*, and D*Mef2*-GAL4 (Supplementary Table S1). We screened for defects during embryonic muscle formation and testes muscle formation. The expression of the miRNA mir-276b (CR42904) and mir-276a (CR43001) showed some muscle defects during embryonic muscle development (Figure 1A,B). However, the overexpression phenotype of mir-276a showed more severe defects (Figure 1C) and displayed additional defects during testis muscle development (Figure 1F,F’). The expression of mir-276a in embryos and testis was confirmed through reverse transcription (RT)-PCR (Figures S1 and S2). Furthermore, flies overexpressing mir-276a were not able to hatch and pharate adult lethal.

Usually, the wild-type testis possesses an elongated coiled-like structure (Figure 1D,E), which is totally covered with a sheath of multinucleated muscles, including the tip of the testis (Figure 1E, asterisk). The testis of mir-276a-expressing males, however, displayed a short testis with a bulky tip (Figure 1F). Furthermore, a closer examination of the muscle sheath revealed that muscles fail to cover the entire testis, and many holes were observed in the testis sheath (Figure 1F,F’ asterisks).

### 3.2. Myoblasts Are Present at the Genital Disk and Express the Transcription Factors Mef2 and Lame Duck

**Figure 2:**
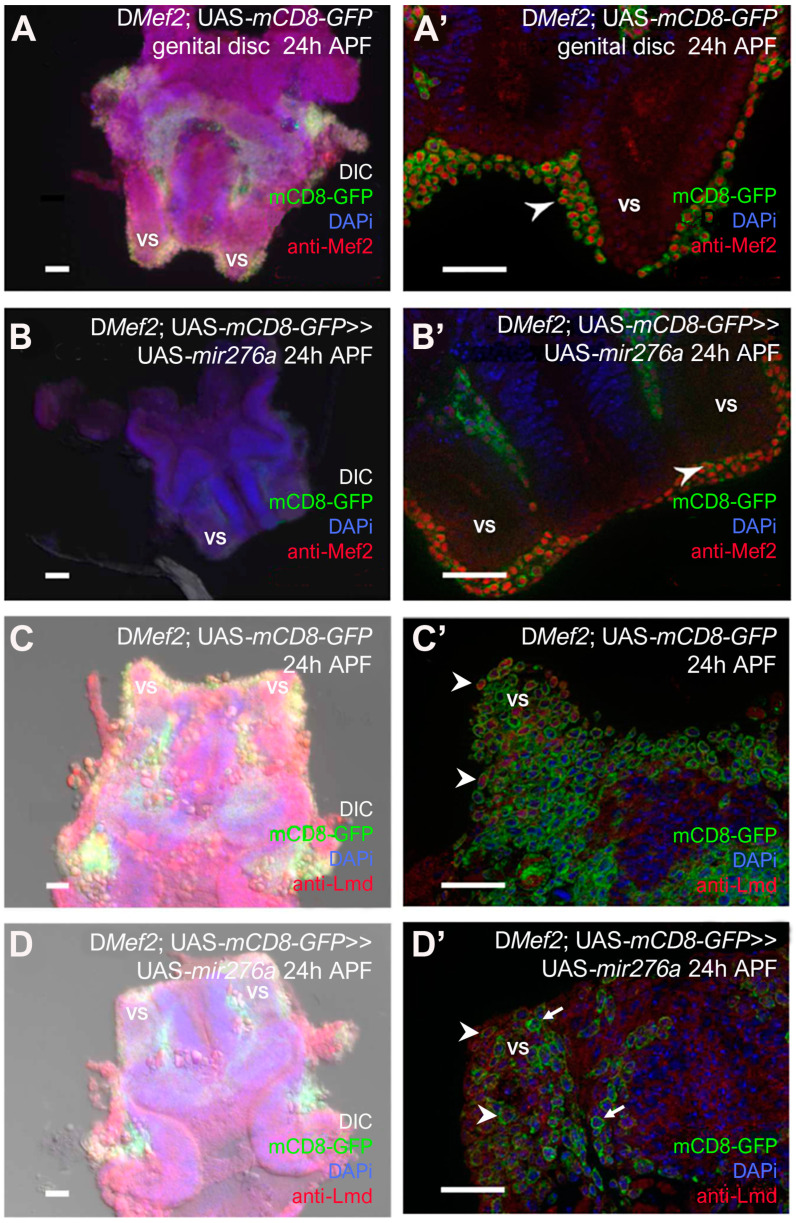
Founder cells and fusion-competent myoblasts are specified: (**A**) Overview of a 24 h APF genital disc expressing UAS-*mCD8*-GFP with D*Mef2*-GAL4 and stained with anti-Mef2 and DAPi. (**A’**) Myoblasts labeled with Mef2 (red, arrowhead). (**B**) Overview of a 24 h APF genital disc co-expressing UAS-*mCD8*-GFP and UAS-*mir276a* with D*Mef2*-GAL4 and stained with anti-Mef2 and DAPi. (**B’**) Myoblasts labeled with Mef2 (red, arrowhead). (**C**) Overview of a 24 h APF genital disc expressing UAS-*mCD8*-GFP with D*Mef2*-GAL4 and stained with anti-Lmd and DAPi. (**C’**) Myoblasts labeled with Lmd (red, arrowheads). (**D**) Overview of a 24 h APF genital disc co-expressing UAS-*mCD8*-GFP and UAS-*mir276a* with D*Mef2*-GAL4 and stained with anti-Lmd and DAPi. (**D’**) Myoblasts labeled with Lmd (red, arrowheads). Arrows mark nascent myotubes. Scale bars 20 μm. vs = seminal vesicles.

The failure of myotubes to cover the testis might be due to a lack of myoblast formation. Mef2 expression is important for adult muscle development. Its transcription is upregulated in whole animals 12 h after puparium formation (APF) at a time when muscle-patterning events occur [34,35]. Mef2-positive myoblasts have been observed at the onset of metamorphosis at the genital disc and start to proliferate during the first hours of metamorphosis [12]. At 24 h APF, the seminal vesicles grow closer to the testes, and the myoblasts that give rise to the testis muscles are clearly visible. To monitor myoblasts at the genital disc, we expressed UAS-*mCD8-GFP* with D*Mef2*-GAL4 in wild-type genital discs (Figure 2A,A’) and UAS-*mir276a* expressing genital discs (Figure 2B,B’). To determine the expression of Mef2, we employed the anti-Mef2 antibody (Figure 2A’,B’). The myoblast nuclei were visualized by performing DAPi staining, and a co-localization with DAPi and Mef2 was expected. We observed the presence of Mef2-positive myoblasts at the prospective seminal vesicles in UAS-*mCD8*-GFP expressing myoblast and in UAS-*mCD8*-GFP and UAS-*mir-276a* co-expressing myoblasts (Figure 2A’,B’, arrowhead). At 24 h APF, the myoblasts had not fused, and nascent myotubes were not visible. The presence of myoblasts indicates that the overexpression phenotype of mir-276a during testis formation is not due to a lack of myoblasts. The detection of the Mef2 protein further indicates that Mef2 is not a target of mir-276a.

Next, we investigated whether fusion-competent myoblasts are specified. The Gli transcription factor Lmd marks exclusively fusion-competent myoblasts during embryonic muscle development and is transcribed in testes myoblasts at 24 h APF [12]. Lmd-positive myoblasts were detected at the prospective seminal vesicles of control genital discs expressing UAS-*mCD8*-GFP with D-*Mef2*-GAL4 (Figure 2C,C’ arrowheads) and in myoblasts co-expressing UAS-*mCD8*-GFP and UAS-*mir276a* with D*Mef2*-GAL4 (Figure 2D,D’ arrowheads). Moreover, we observed the formation of nascent myotubes at the prospective seminal vesicles of co-expressing UAS-*mCD8-GFP* and UAS-*mir276a* myoblasts. These data suggest that fusion-competent myoblasts are specified correctly when mir-276a is overexpressed and that the *lmd* mRNA is not a target of mir-276a. In addition, mir-276a-expressing myoblasts are able to fuse (Figure 2D’, arrows).

Collectively, these data led us to conclude that the overexpression of mir-276a does not interfere with the specification of founder cells and fusion-competent myoblasts in the region of the prospective seminal vesicles. Moreover, nascent myotubes at the prospective seminal vesicles imply that the overexpression phenotype of UAS-*mir276a* (Figure 1F,F’) is not due to a failure in myoblast fusion.

### 3.3. mir276a Binds to the 3′UTR of Heartless to Downregulate the Heartless mRNA

**Figure 3:**
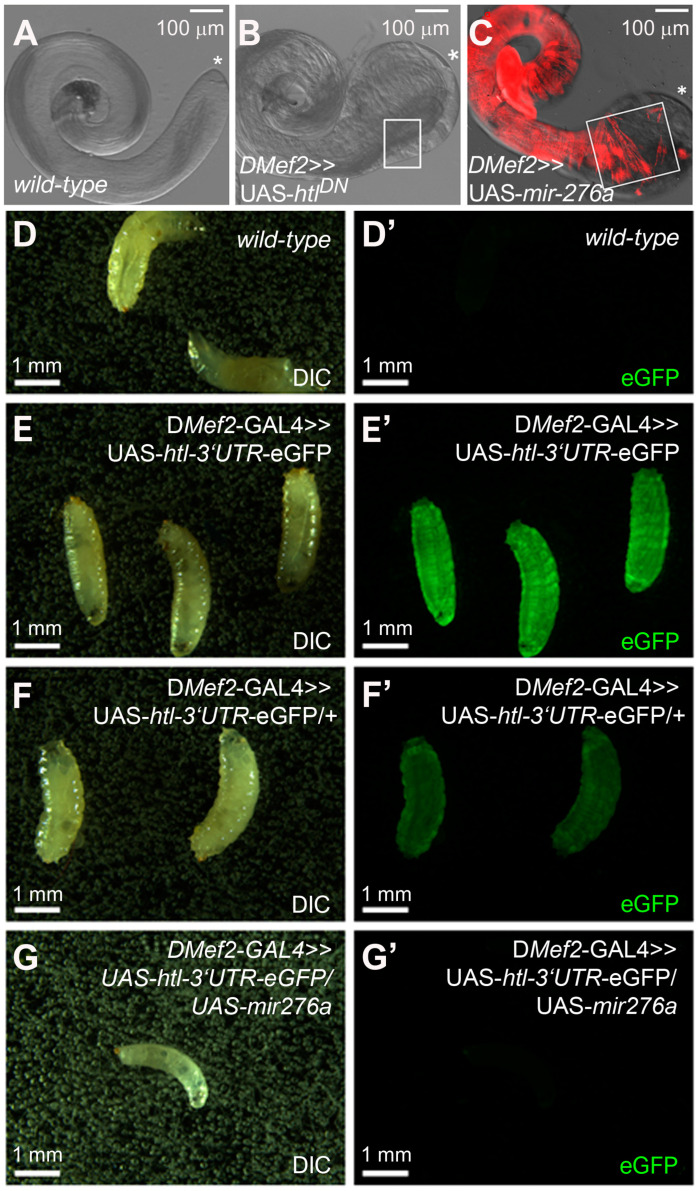
GFP sensor assay to show that *heartless* is an in vivo target of mir-276a: (**A**–**C**) Analysis of adult testes dissected from *Drosophila* males. The framed areas show how far testis muscles colonize the testis. The asterisk marks the tip of the testis. (**A**) Wild type. (**B**) Dominant-negative UAS-*htl* expressed with D*Mef2*-GAL4. (**C**) Expression of UAS-*mir276a* with D-*Mef2*-GAL4 and stained with Phalloidin-TRITC. (**D–G**) GFP sensor assay. (**D**,**D’**) Wild-type, third-instar larvae express no eGFP. (**E**,**E’**) Recombinant third-instar larvae carrying two copies of D*Mef2*-GAL4 >> UAS-*htl*-3′UTR-eGFP with a strong eGFP expression. (**F**,**F’**) Recombinant third-instar larvae carrying one copy of D*Mef2*-GAL4 >> UAS-*htl*-3′UTR-eGFP with detectable eGFP expression. (**G**,**G’**) Recombinant third-instar larva carrying one copy of D*Mef2*-GAL4 >> UAS-*htl*-3′UTR-eGFP and one copy of UAS-*mir276a* express no eGFP.

During testes formation, myoblasts fuse to form multinucleated myotubes that migrate onto the pupal testis when the prospective seminal vesicles and testes grow towards each other and unite. The disturbance of myotube migration results in a failure of coiled-like testis formation [13,14,15,16]. The loss of the FGF receptor *heartless* (*htl*), its intracellular adaptor protein *stumps*, and *downstream of FGF* (*dof*) are essential for proper myotube migration [13]. The expression of Htl lacking the kinase domain with D*Mef2*-GAL4 phenocopies the bulky-tip phenotype of D*Mef2*-GAL4 >> UAS-*mir276a* (Figure 3B,C). Based on similar phenotypes, we hypothesized that the 3′UTR of the *htl* transcript might be a target of mir-276a. Moreover, *htl* is a predicted target of mir-276a-5p (TargetScanFly 7.2).

To investigate whether mir-276a targets the 3′UTR of *htl*, a GFP sensor assay was performed. For this assay, the 3′UTR of the *htl* cDNA LD32130 was amplified using PCR and cloned into the pUASt-attB-rfa-eGFP vector [29] and injected into *Drosophila* embryos to establish transgenic flies. Using meiotic recombination, we establish a homozygous D*Mef2*-GAL >> UAS-*htl-3′UTR*-eGFP fly strain. If *htl* is a target of mir-276a, we expected the downregulation of eGFP in animals co-expressing UAS-*mir-276a* with the putative eGFP sensor. The expression of eGFP was examined in the body wall musculature of third-instar larvae. Wild-type third-instar larvae have no eGFP expression (Figure 3D’). We found that homozygous larvae carrying two copies of D*Mef2*-GAL >> UAS-*htl-3′UTR*-eGFP displayed a strong eGFP expression (Figure 3E’). The eGFP expression was reduced in the third-instar larvae of D*Mef2*-GAL >> UAS-*htl-3′UTR*-eGFP animals that were crossed to wild-type animals and carried only one copy of D*Mef2*-GAL >> UAS-*htl-3′UTR*-eGFP (Figure 3F’). However, eGFP was clearly detectable in those larvae. The co-expression of mir-276a with UAS-*htl-3′UTR*-eGFP caused a complete downregulation of eGFP (Figure 3G’), confirming the notion that mir-276a targets the 3′UTR of the FGF receptor mRNA *htl*. A downregulation of UAS-*htl-3′UTR*-eGFP was also achieved when UAS-*mir276b* was co-expressed with D*Mef2*-GAL4.

### 3.4. mir-276a Overexpressing Indirect Flight Muscles Show Severe Defects

**Figure 4:**
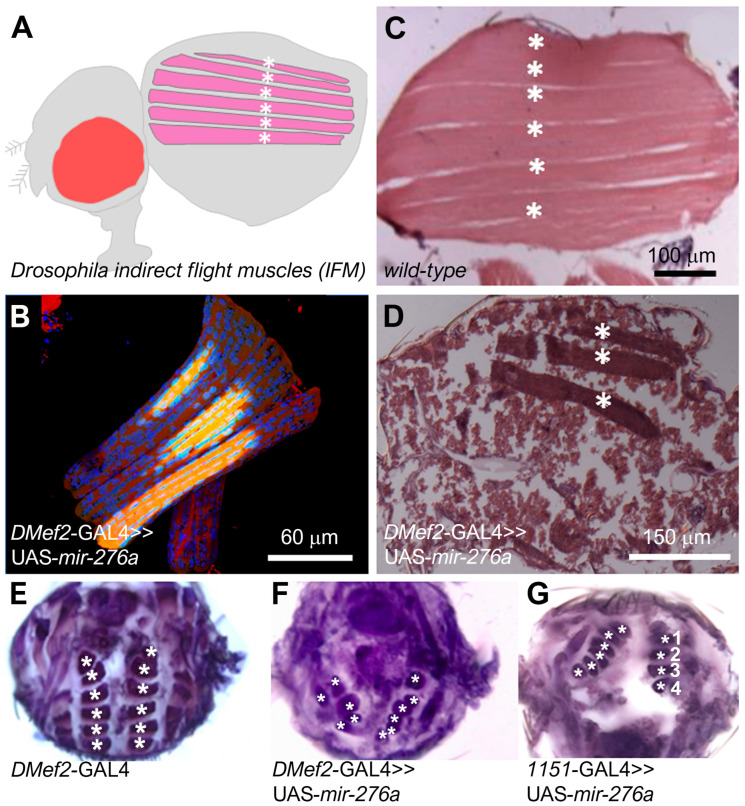
The number of indirect flight muscles is reduced in UAS-*mir-276a*-expressing animals: (**A**) Longitudinal view. Scheme of the six indirect flight muscles in the *Drosophila* adult. (**B**) Confocal image of mir-276a overexpressing DLMs stained with Phalloidin and DAPi. Only three DLMs are seen. Scale bar 60 μm. (**C**) Longitudinal paraffin section of an adult wild-type fly stained with hematoxylin and eosin. Six DLM muscles are visible (asterisks). Scale bar 100 μm. (**D**) Longitudinal paraffin section of an adult fly expressing UAS-*mir-276a* in myoblasts with D*Mef2*-GAL4. Three DLMs are detectable (asterisks). Scale bar 150 μm. (**E**–**G**) Transverse sections of indirect flight muscles of adult flies stained with hematoxylin. 10× Objective. (**E**) D*Mef2*-GAL4 fly with a normal number of indirect flight muscles (asterisks) on each site of the thorax. (**F**) Adult fly expressing UAS-*mir276a* in myoblasts, showing an abnormal size and the number of indirect flight muscles (asterisks). (**G**) Adult fly expressing UAS-*mir276a* only in adult myoblasts with *1151*-GAL4, showing an abnormal size and a number of indirect flight muscles (asterisks). On the right side only four DLMs are detectable.

The finding that flies expressing mir-276a with D*Mef2*-GAL4 are pharate lethal and unable to hatch prompted us to investigate whether adult muscle formation is defective in these flies. To assess whether myogenesis proceeds properly in these flies, we performed a histological analysis of the major thoracic indirect flight muscles, the DLMs (Figure 4A). Confocal images (Figure 4B) and longitudinal sections (Figure 4C,D) first suggested that UAS-*mir-276a*-expressing flies exhibit a template splitting defect. To verify these findings, we performed transverse sections (Figure 4E,F). In wild-type flies, six pairs of DLMs are visible on each side of the thorax (Figure 4E, asterisks). However, DLMs in UAS-*mir-276a*-overexpressing flies are less organized in comparison to wild-type flies, and the number of DLMs is reduced (Figure 4F, asterisks). D*Mef2*-GAL4 drives the expression of UAS-*mir-276a* during embryonic and adult development. To determine whether mir-276a specifically affects adult myogenesis, we used the *1151*-GAL4 driver line that is active in adult myoblasts 18 h before pupal formation [17,36]. Flies expressing UAS-*mir276a* with *1151*-GAL4 showed DLMs, but as with D*Mef2*-GAL4, we observed a reduction in the number of DLMs. Altogether, we found four to six DLMs. Overall, these observations indicate that larval template muscles and myoblasts are normally specified.

**Figure 5:**
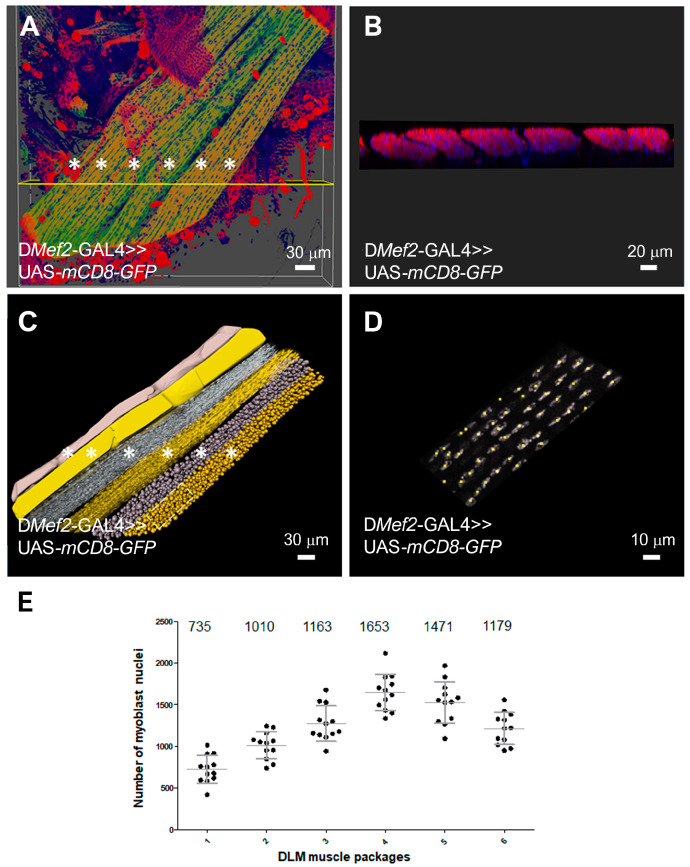
Determination of nuclei for each DLM muscle: (**A**) Three-dimensional view of the six DLM muscles (asterisks) marked with UAS-*mCD8*-GFP, DAPi, and Phalloidin. Scale bar 30 μm. (**B**) Cross-section of the flight muscles that are highlighted by the yellow line and the asterisks in (**A**). Scale bar 20 μm. (**C**) Illustration of the individual work steps for quantifying the cell nuclei of the six DLMs marked by asterisks. Scale bar 30 μm. (**D**) Illustration of the accuracy of the Imaris 9.3 software in the counting of nuclei. Higher magnification of the area marked in (**C**). Scale bar 10 μm. (**E**) Quantification of the nuclei in the DLMS.

Myoblast fusion is a prerequisite for the splitting of template muscles. DLMs are large multinucleated cells that contain thousands of nuclei. To quantify whether mir-276a contains fewer nuclei, we first established a method to analyze the number of nuclei within the six DLMs in wild-type pupae. For the quantification of nuclei, we dissected 40 h APF pupae as described by Weitkunat and Schnorrer [32]. Pupae that expressed UAS-*mCD8*-eGFP with D*Mef2*-GAL4 were stained with Phalloidin and DAPi. DLMs were imaged using a Leica Sp8 confocal microscope (Figure 5A,B) and analyzed using the Imaris Image Analysis software 9.3 (Figure 5C,D, Video 1). With the help of the Imaris “Surface module”, the individual DLMs were segmented by manually outlining each individual muscle (Figure 5C). Next, the “spots module” was used to count the nuclei automatically (Figure 5E). Figure 5D shows how the nuclei are identified and replaced by the software. Each nucleus is replaced by a yellow square (see also Video 1). This approach revealed that the first DLM muscle is the smallest, with approximately 735 nuclei. The second muscle contains around 1010, the third 1163, the fourth 1653, the fifth 1471, and the six 1179 nuclei (Figure 5E).

Next, we attempted to determine the nuclei number of DLMs from UAS-*mir-276a* expressing animals. But, since DLMs are disorganized (Figure 4F), the preparation of DLMs was very difficult, as the muscle easily disintegrated during preparation, and in most cases, we only obtained three DLMs, as shown in Figure 4B,D. For this reason, we failed to determine the number of nuclei in UAS-*mir276a*-expressing flies. Since we identified *heartless* as a target of mir-276a during testis muscle migration, we asked the question of whether *heartless* plays a role in indirect flight muscle formation. To characterize the role of *heartless* in DLM formation, we used an RNAi-mediated approach and analyzed whether the loss of *heartless* phenocopies the mir-276a phenotype.

### 3.5. The Loss of the Heartless Pathway Reduces DLM Size, but the Number of DLM Muscles Is Not Reduced

**Figure 6:**
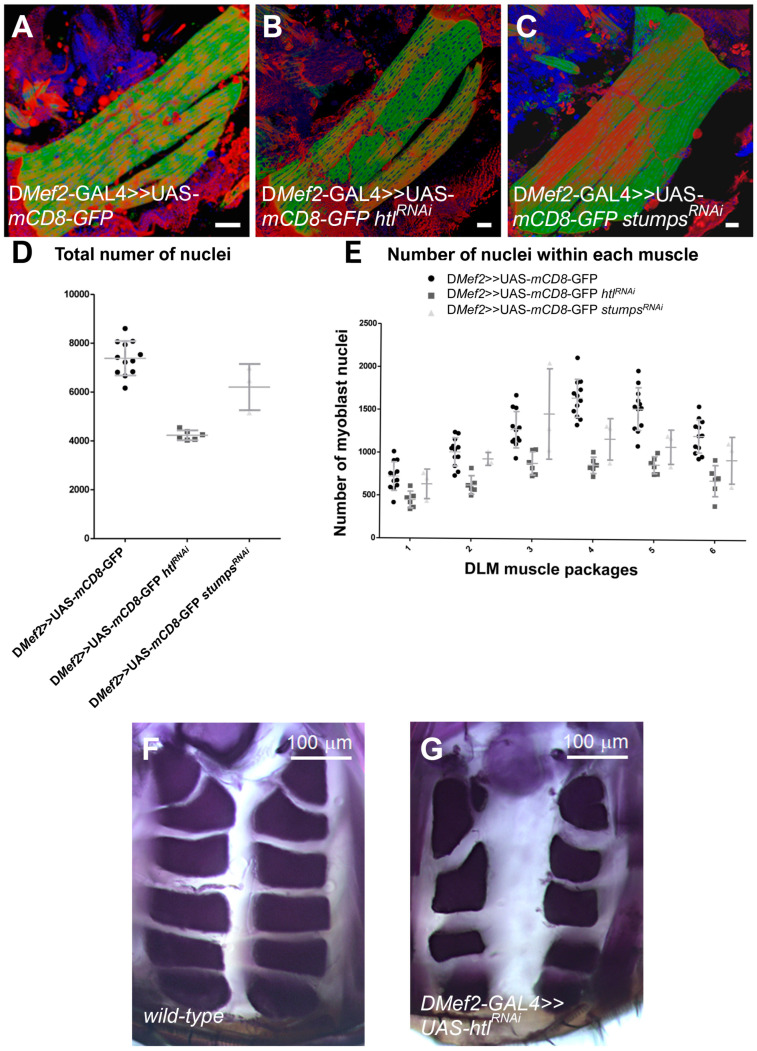
RNAi-mediated knockdown of heartless and stumps in DLMs: Three-dimensional view of (**A**) *wild-type* DLMs expressing UAS-*mCD8*-GFP with D*Mef2*-GAL4, (**B**) UAS-*htl*-RNAi expressing DLMs with D*Mef2*-GAL4, and (**C**) UAS-*stumps*-RNAi expressing DLMs with D*Mef2*-GAL4. (**D**) Total number of nuclei in DLMs from D*mef2* >> UAS-*mCD8*-eGFP, D*mef2* >> UAS-*htl*-RNAi, and D*Mef2* >> UAS-*stumps*-RNAi flies. (**E**) Number of nuclei within each DLM muscle for the six DLM muscles of D*Mef2* >> UAS-*mCD8*-eGFP, D*Mef2* >> UAS-*htl*-RNAi, and D*Mef2* >> UAS-*stumps*-RNAi. (**F**,**G**) Transverse sections of DLMs. (**F**) Wild type. (**G**) D*Mef2*-GAL4 >> UAS-*htl*-RNAi. Scale bars in A–C 30 μm, scale bars in F and G 100 μm.

To determine whether the knockdown of Heartless leads to similar defects like mir-276a, we expressed UAS-RNAi lines of the FGF receptor *heartless* and its intracellular adaptor *stumps* in muscles using D*Mef2*-GAL4 (Figure 6A–C). Thereafter, we compared the phenotypes of these animals with only UAS-*mCD8*-GFP-expressing flies. To quantify the number of nuclei in the DLMs, we used the Imaris software, as shown in Figure 5. The total number of nuclei was clearly reduced in UAS-*htl*-RNAi and UAS-*stumps*-RNAi-expressing adult flies (Figure 6D). A reduction in the number of nuclei was further confirmed when we compared the individual DLM muscles with each other (Figure 6E). However, in the transversal section, the arrangement of DLM muscles was comparable to UAS-*mCD8*-GFP-expressing adults. A severe reduction in the number of DLM muscles, as observed in mir-276a expressing flies (Figure 4F,G), was not observed.

Taken together, these data confirm that *heartless* is required to increase the size of DLMs through myoblast fusion. Nonetheless, the splitting of muscles is not as severely impaired as in mir-276a overexpressing adults, suggesting that the *heartless* mRNA is not a predominant target of mir-276a during flight muscle development.

## 4. Discussion

The impairment of muscle formation and function has a dramatic impact on the quality of life. Accordingly, muscle formation, muscle growth, and repair must be strictly regulated. Mature miRNAs are associated with Argonaute (AGO) proteins, which facilitate their function in gene silencing at the post-transcriptional level [37]. Comparable to transcription factors, microRNAs can affect the transcript and protein levels of hundreds of targets in animals and, therefore, form complex regulatory networks [38]. Thus, the diversity of miRNAs and their ability to modulate gene expression make them powerful agents of fine-tuning control in various cellular functions.

In this study, we show for the first time that mir-276a is required for muscle development and identified the FGF receptor *heartless* as a target for mir-276a. So far, mir-276a has been identified as an abundant miRNA in fly heads, where it regulates the clock gene *timeless* [39]. Furthermore, it is rhythmically oscillating in the heads of wild-type flies and downregulates *neuropeptide F receptor 1* (*npfr1*) [40]. mir-276a expression has been detected in mushroom bodies, the pars intercerebralis, lateral dorsal neurons, and the subpharyngeal ganglion [40]. mir-276a is further required for long-term memory by controlling *Dopamine receptor* expression (*Dop1R1* and *Dop1R2*) [41]. In larval sensory neurons, mir-276a functions together with the Fragile X mental retardation protein (FMR1) in space-filling dendrite morphogenesis by regulating the transcription of the H3K18 acetyltransferase enzyme *nejire* [42]. Although mir276a has important neuronal functions, it is not conserved in mammals. Nevertheless, it is conserved in insects and forms a family together with mir-276b. The family members mir-276a-5p and mir-276a3p are both expressed in 0–24 h embryos in third-instar larvae and pupae [43]. Using RT-PCR, we could confirm the expression of mir-276a in embryos and detected mir-276a in RNAs isolated from testes and thorax muscles.

We found that the muscle-specific overexpression of mir-276a causes defects in different aspects of muscle development. During embryonic muscle development, we observed missing larval body wall muscles. But, the overexpression of mir-276a has an additional impact on adult myogenesis. In indirect flight muscle development, the six dorsal longitudinal muscles (DLMs) at the thorax are displaced, and their number is reduced, suggesting that template muscle splitting is impaired. During adult testis muscle development, the overexpressing of mir-276a in nascent myotubes impairs myotube migration, which results in testis shaping defects.

But how does the overexpression of mir-276a regulate all these different aspects of muscle formation? mir-276a-3p, mir-276a-5p, and mir-276b-5p have been shown to be ecdysone-responsive miRNAs [44,45]. In a recent study, ecdysone was demonstrated to promote the fusion of embryonic myoblasts by activating the transcription of *antisocial/rolling pebbles* [46]. Antisocial/Rolling pebbles is a founder cell-specific adaptor that binds to the cell adhesion protein Dumbfounded to transduce the fusion signal from the myoblast membrane into a cellular response. In this study, embryos carrying defects in genes encoding enzymes required for ecdysone biogenesis displayed unfused myoblasts [46]. Myoblast fusion is a highly dynamic process in which multiple fusion-competent myoblasts can fuse with a single growing myotube. This circumstance requires the precise regulation of fusion proteins during the fusion of fusion-competent myoblasts with growing myotubes. The overexpression of mir-276a during embryonic development does not phenocopy the myoblast fusion phenotype of homozygous *heatless* loss-of-function mutants. However, as shown in neurons, mir-276a can affect many transcripts. It is, therefore, conceivable that mir-276a silences additional targets besides *heartless* during myoblast fusion. It is also possible that mir-276a modulates histone modifications together with other RNA-binding proteins, such as FMR1 [42]. In situ hybridizations have detected the FMR1 transcript in somatic muscles during embryonic stage 12 at the onset of myoblast fusion [47].

The overexpression of mir-276a also affects dorsal longitudinal muscle formation. The number of DLMs was reduced when mir-276a expression was driven with D*Mef2*- and *1151*-GAL4. These findings indicate that the overexpression of mir-276a inhibits the splitting of the larval longitudinal muscles (LOMs) that escape histolysis during metamorphosis. Recently, it was shown that mir-276a is upregulated during larval-to-pupae transition [44]. The overexpression of mir-276a in the larval fat body leads to a decrease in the larval body weight, a reduction in wing cell size, and a suppression of cell proliferation [45]. This might lead to a proliferation defect of the AMP pool and, thus, to a reduced number of myoblasts, leading to LOM splitting defects in D*Mef2*-GAL4 >> UAS-*mir-276a*-overexpressing flies. A reduced myoblast pool size has also been demonstrated in the absence of FGF signaling [22]. We could confirm that D*Mef2*-GAL4 >> UAS-*htl^RNAi^*-expressing flies possess a reduced number of nuclei in DLMs. However, we did not observe any LOM splitting defects. The formation of the post-mitotic myoblast pool during DLM formation takes place in two phases. The first proliferation phase is initiated 24 to 48 h after egg lay (AEL) by Notch and Serrate signaling. The second proliferation phase takes place 72 h AEL through the activation of the Wnt-β-Catenin signaling pathway [17,21]. Heartless appears to be involved in the second phase by activating β-catenin [22]. The different phenotypes between mir-276a and *heartless* during flight muscle development may indicate that mir-276a is important during the first phase of proliferation, while *heartless* is required for the second phase.

We further observed that the overexpression of mir-276a with D*Mef2*-GAL4 causes testis shaping defects. The expression of Mef2 in adult myoblasts depends on ecdysone and the ecdysone-induced Borad Complex (BR-C) [36]. Since the transcriptional activation of mir-276a involves the ecdysone receptor EcR and BR-C, we have investigated the expression of Mef2 in myoblasts at the prospective seminal vesicles. However, Mef2 continues to be expressed in myoblasts after overexpression of mir-276a, as does the fusion-competent transcription factor Lame duck. The *heartless* 3′UTR is a predicted target for the less conserved family of mir-276a-5p. By using an in vivo GFP sensor assay, we demonstrate that mir-276a silences GFP expression by binding to the 3′UTR of *heartless*. Heartless is like mir-276a, which is required for the migration of nascent myotubes and displays a similar phenotype [13]. The collective migration of myotubes from the genital disc onto the testis has been shown to be crucial for testis shaping [13,14,15]. In particular, the *heartless*-RNAi-mediated knockdown or the expression of dominant-negative *heartless* leads to a partial cover of the testis with muscles and to the loss of the coiled testis shape [13]. Thus, nascent myotube migration and testis shaping are impaired by the mir-276a-dependent silencing of *heartless*.

## Data Availability

Data are contained within the article and Supplementary Materials.

## Supplementary Materials

The following supporting information can be downloaded at: https://www.mdpi.com/article/10.3390/cells14050368/s1, Video S1: Nuclei counting using Imaris. Figure S1: Transcription of mir-276a; Figure S2: Extracted RT-PCR fragments, Table S1: Lethality Screen of UAS-*mir-RNAs* crossed to *DMef2*-GAL4 or UAS-*Dcr2*; *DMef2*-GAL4.

## References

[1] Baylies M.K., Bate M. (1996). Twist: A myogenic switch in *Drosophila*. Science.

[2] Carmena A., Bate M., Jiménez F. (1995). Lethal of scute, a proneural gene, participates in the specification of muscle progenitors during *Drosophila* embryogenesis. Genes Dev..

[3] Bate M., Martinez Arias A. (1991). The embryonic origin of imaginal discs in *Drosophila*. Development.

[4] Laurichesse Q., Soler C. (2020). Muscle development: A view from adult myogenesis in *Drosophila*. Semin. Cell Dev. Biol..

[5] Ruiz-Gómez M., Coutts N., Price A., Taylor M.V., Bate M. (2000). *Drosophila* dumbfounded: A myoblast attractant essential for fusion. Cell.

[6] Strünkelnberg M., Bonengel B., Moda L.M., Hertenstein A., de Couet H.G., Ramos R.G., Fischbach K.F. (2001). rst and its paralogue kirre act redundantly during embryonic muscle development in *Drosophila*. Development.

[7] Duan H., Skeath J.B., Nguyen H.T. (2001). *Drosophila* Lame duck, a novel member of the Gli superfamily, acts as a key regulator of myogenesis by controlling fusion competent myoblast development. Development.

[8] Ruiz-Gomez M., Coutts N., Suster M.L., Landgraf M., Bate M. (2002). Minc function in *Drosophila* myogenesis. Development.

[9] Rout P., Preußner M., Önel S.F. (2022). *Drosophila melanogaster*: A Model System to Study Distinct Genetic Programs in Myoblast Fusion. Cells.

[10] Boukhatmi H. (2021). *Drosophila*, an Integrative Model to Study the Features of Muscle Stem Cells in Development and Regeneration. Cells.

[11] Ahmad S.M., Baker B.S. (2002). Sex-specific deployment of FGF signaling in *Drosophila* recruits mesodermal cells into the male genital imaginal disc. Cell.

[12] Susic-Jung L., Hornbruch-Freitag C., Kuckwa J., Rexer K., Lammel U., Renkawitz-Pohl R. (2012). Multinucleated smooth muscles and mononucleated as well as multinucleated striated muscles develop during establishment of the male reproductive organs of *Drosophila melanogaster*. Dev. Biol..

[13] Kuckwa J., Fritzen K., Buttgereit D., Rothenbusch-Fender S., Renkawitz-Pohl R. (2016). A new level of plasticity: Drosophila smooth-like testes muscles compensate failure of myoblast fusion. Development.

[14] Rothenbusch-Fender S., Fritzen K., Bischoff M.C., Buttgereit D., Önel S.F., Renkawitz-Pohl R. (2017). Myotube migration to cover and shape the testis of *Drosophila* depends on Heartless, Cadherin/Catenin, and myosin II. Biol. Open.

[15] Bischoff M.C., Lieb S., Renkawitz-Pohl R., Bogdan S. (2021). Filopodia-based contact stimulation of cell migration drives tissue morphogenesis. Nat. Commun..

[16] Bischoff M.C., Norton J.E., Munguia E.A., Clark S.E., Gurley N.J., Korankye R., Gyabaah E.A., Encarnacion T., Serody C.J., Jones C.D. (2025). A large reverse-genetic screen identifies numerous regulators of testis nascent myotube collective cell migration and collective organ sculpting. Mol. Biol. Cell.

[17] Gunage R.D., Dhanyasi N., Reichert H., VijayRaghavan K. (2017). Drosophila adult muscle development and regeneration. Semin. Cell Dev. Biol..

[18] Roy S., VijayRaghavan K. (1998). Patterning Muscles Using Organizers: Larval Muscle Templates and Adult Myoblasts Actively Interact to Pattern the Dorsal Longitudinal Flight Muscles of *Drosophila*. J. Cell Biol..

[19] Anant S., Roy S., VijayRaghavan K. (1998). Twist and Notch negatively regulate adult muscle differentiation in *Drosophila*. Development.

[20] Bernard F., Dutriaux A., Silber J., Lalouette A. (2006). Notch pathway repression by vestigial is required to promote indirect flight muscle differentiation in *Drosophila melanogaster*. Dev. Biol..

[21] Gunage R.D., Reichert H., VijayRaghavan K. (2014). Identification of a new stem cell population that generates *Drosophila* flight muscles. eLife.

[22] Vishal K., Lovato T.L., Bragg C., Chechenova M.B., Cripps R.M. (2020). FGF signaling promotes myoblast proliferation through activation of wingless signaling. Dev. Biol..

[23] Gildor B., Schejter E.D., Shilo B.Z. (2012). Bidirectional Notch activation represses fusion competence in swarming adult *Drosophila* myoblasts. Development.

[24] Bartel D. (2018). Metazoan MicroRNAs. Cell.

[25] Paul P., Chakraborty A., Sarkar D., Langthasa M., Rahman M., Bari M., Singha R.S., Malakar A.K., Chakraborty S. (2018). Interplay between miRNAs and human diseases. J. Cell. Physiol..

[26] Bour B.A., O’Brien M.A., Lockwood W.L., Goldstein E.S., Bodmer R., Taghert P.H., Abmayr S.M., Nguyen H.T. (1995). *Drosophila* MEF2, a transcription factor that is essential for myogenesis. Genes. Dev..

[27] Lilly B., Zhao B., Ranganayakulu G., Paterson B.M., Schulz R.A., Olson E.N. (1995). Requirement of MADS domain transcription factor D-MEF2 for muscle formation in *Drosophila*. Science.

[28] Ranganayakulu G., Schulz R.A., Olson E.N. (1996). Wingless signaling induces Nautilus expression in the ventral mesoderm of the *Drosophila* embryo. Dev. Biol..

[29] Gohl C., Banovic D., Grevelhörster A., Bogdan S. (2010). WAVE forms hetero- and homo-oligomeric complexes at integrin junctions in Drosophila visualized by bimolecular fluorescence complementation. J. Biol. Chem..

[30] Buttgereit D., Paululat A., Renkawitz-Pohl R. (1996). Muscle development and attachment to the epidermis is accompanied by expression of beta 3 and beta 1 tubulin isotypes, respectively. Int. J. Dev. Biol..

[31] Kucherenko M.M., Marrone A.K., Rishko V.M., Yatsenko A.S., Klepzig A., Shcherbata H.R. (2010). Paraffin-Embedded and Frozen Sections of *Drosophila* Adult Muscles. J. Vis. Exp..

[32] Weitkunat M., Schnorrer F. (2014). A guide to study *Drosophila* muscle biology. Methods.

[33] Schertel C., Rutishauser T., Förstemann K., Basler K. (2012). Functional characterization of *Drosophila* microRNAs by a novel in vivo library. Genetics.

[34] White K.P., Rifkin S.A., Hurban P., Hogness D.S. (1999). Microarray analysis of Drosophila development during metamorphosis. Science.

[35] Fernandes J., Bate M., Vijayraghavan K. (1991). Development of the indirect flight muscles of Drosophila. Development.

[36] Lovato T.L., Benjamin A.R., Cripps R.M. (2005). Transcription of Myocyte enhancer factor-2 in adult *Drosophila* myoblasts is induced by the steroid hormone ecdysone. Dev. Biol..

[37] Shang R., Lee S., Senavirathne G., Lai E.C. (2023). microRNAs in action: Biogenesis, function and regulation. Nat. Rev. Genet..

[38] Panni S., Lovering R.C., Porras P., Orchard S. (2020). Non-coding RNA regulatory networks. Biochim. Biophys. Acta Gene Regul. Mech..

[39] Chen X., Rosbash M. (2016). mir-276a strengthens *Drosophila* circadian rhythms by regulating timeless expression. Proc. Natl. Acad. Sci. USA.

[40] Zhang R., Zhao X., Du J., Wei L., Zhao Z. (2021). Regulatory mechanism of daily sleep by miR-276a. FASEB J..

[41] Li W., Cressy M., Qin H., Fulga T., Van Vactor D., Dubnau J. (2013). MicroRNA-276a functions in ellipsoid body and mushroom body neurons for naive and conditioned olfactory avoidance in *Drosophila*. J. Neurosci..

[42] Li H., Gavis E.R. (2022). *Drosophila* FMRP controls miR-276-mediated regulation of nejire mRNA for space-filling dendrite development. G3 Genes|Genomes|Genetics.

[43] Okamura K., Phillips M.D., Tyler D.M., Duan H., Chou Y.T., Lai E.C. (2008). The regulatory activity of microRNA* species has substantial influence on microRNA and 3′ UTR evolution. Nat. Struct. Mol. Biol..

[44] Lim D.H., Lee S., Han J.Y., Choi M.S., Hong J.S., Seong Y., Kwon Y.S., Lee Y.S. (2018). Ecdysone-responsive microRNA-252-5p controls the cell cycle by targeting Abi in *Drosophila*. FASEB J..

[45] Lee S., Kim N., Jang D., Kim H.K., Kim J., Jeon J.W., Lim D.H. (2023). Ecdysone-induced microRNA miR-276a-3p controls developmental growth by targeting the insulin-like receptor in *Drosophila*. Insect Mol. Biol..

[46] Ruan Z.R., Yu Z., Xing C., Chen E.H. (2024). Inter-organ steroid hormone signaling promotes myoblast fusion via direct transcriptional regulation of a single key effector gene. Curr. Biol..

[47] Schenck A., van de Bor V., Bardoni B., Giangrande A. (2002). Novel features of dFMR1, the *Drosophila* orthologue of the fragile X mental retardation protein. Neurobiol. Dis..

