# Supplementary material for: mir-276a Is Required for Muscle Development in Drosophila and Regulates the FGF Receptor Heartless During the Migration of Nascent Myotubes in the Testis
Source: Cells. 2025 Mar 3;14(5):368. doi: 10.3390/cells14050368 (PMC11898445; doi:10.3390/cells14050368)
Supplement: Supplementary file 1 [file cells-14-00368-s001.zip › cells-3428801-supplementary.pdf]

Supplemental information

We determined the expression of mir-276a in embryos, testis and thorax muscles using reverse transcriptase (RT)-PCR analysis. The pre-mir-276a RNA contains 98 bp and has been shown to be expressed in *Drosophila* S2 cells [40]. We isolated total RNA from embryos of all stages, from isolated testes and from thorax muscles and synthesized a cDNA via reverse transcription. As a control we used water. We obtained several bands after the RT-PCR (Figure S1), which were extracted after gel electrophoresis and sequenced (Figure S2). The sequence of the upper bands in Figure S1 (arrow) mapped to the mir-276a gene locus in Flybase. These data confirm previous results that have demonstrated the transcription of mir-276a-3p and mir-276a-5p in 0-24h embryos, in third instar larvae and in mixed pupae using northern blot analysis [43].

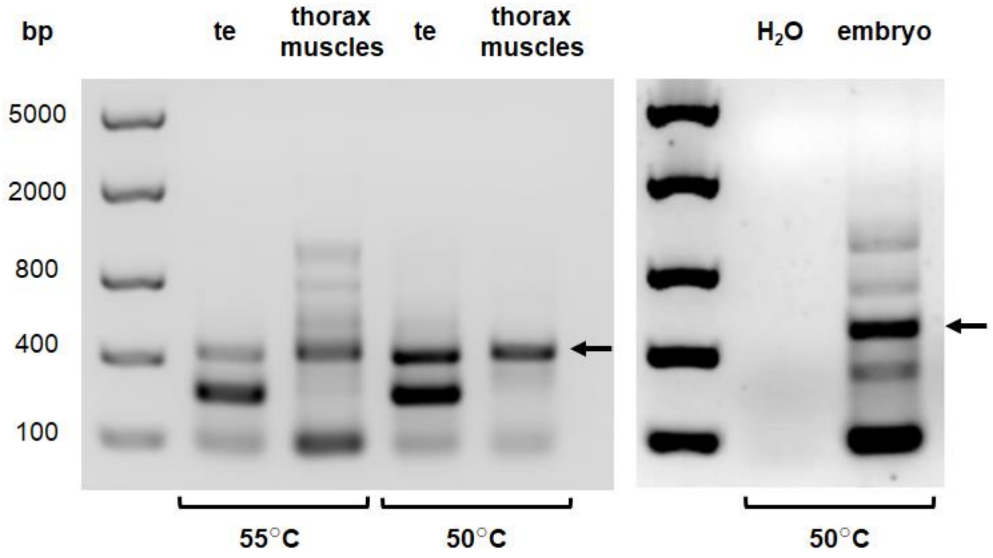

Figure S1 Transcription of mir-276a. The expression of mir-276a in embryos, testis and thorax muscles was determined by performing RT-PCR. Total RNA was extracted from stage 1 to 17 embryos, from adult testes and thorax muscles and transcribed into a cDNA. The obtained RT-PCR products were extracted from the agarose gels and sequenced.

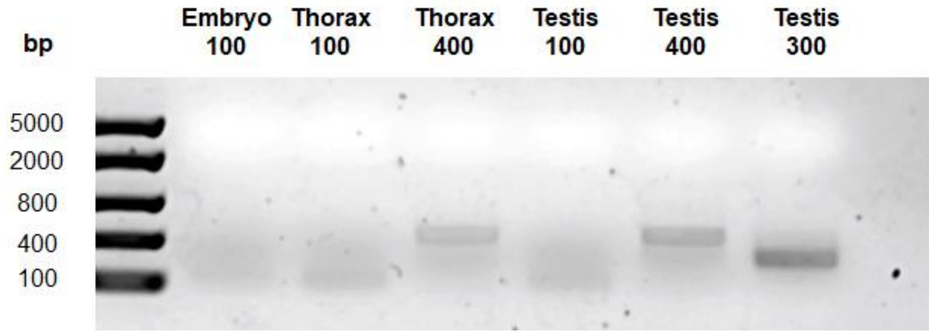

Figure S2 Extracted RT-PCR fragments. 2 µl of the extracted PCR fragments were loaded onto an agarose gel. 5 ng of the PCR fragments were sequenced with mir-276a_f and mir-276a_rev. The 400 bp fragments mapped to the mir-276a gene locus using the BLAST search tool from Flybase.

Method

RNA isolation and RT-PCR

To determine the transcription of mir-276a in embryos, testes and dorsal flight muscles, fertilized eggs of all stages were collected for 2 days. In addition, 10 testis pairs as well as 20 thorax muscles were isolated. RNA isolation was performed using the GE Healthcare illustra RNAspin Mini Isolation Kit (Chalfont St Giles, England) according to the manufacturer’s instructions. For reverse transcription, MuLV Reverse transcriptase (Thermo Fischer Scientific, Waltham, MA, USA) was used and PCRs including 28 (embryos) and 38 (testes and thorax muscles) cycles were performed. We used the following primers to amplify the mir-276a pre-mRNA: mir-276a_f CCT GGT TTT TGC CAT CAG C, mir276a_rev CTT GGT TGT TTT TTG GTC T.

PCR products were analyzed by agarose gel electrophoresis and imaged using the Odyssey Fc Imaging System (LI-COR Bioscience, Lincoln, NE, USA). The GeneJET Gel Extraction Kit from Thermo Scientific was used to purify DNA fragments. A Blast Search in Flybase revealed that the sequence maps to the mir-276a gene locus. The RT-PCRs were repeated three times.

Tabel S1 Lethality Screen of UAS-*mir-RNAs* crossed to *DMef2*-GAL4 or UAS-*Dcr2*; *DMef2*-GAL4

	**UAS-*mirRNA***	**Lethality with *DMef2*-GAL4**	**Lethality with UAS*Dcr-2;* D*Mef2*-GAL4**		**UAS-*mirRNA***	**Lethality with *DMef2*-GAL4**	**Lethality with UAS*Dcr-2;* D*Mef2*-GAL4**
1	mir-1	no	no	55	mir-304	no	no
2	mir-2a-1	no	no	56	mir-306	no	no
3	mir-2a-2	no	no	57	mir-307a	no	no
4	mir-2b-2	no	no	58	mir-307b	no	no
5	mir-2c	no	no	59	mir-308	no	no
6	mir-4	no	no	60	mir-309	no	no
7	mir-5	no	no	61	mir-310	no	no
8	mir-6-1	no	no	62	mir-311	no	no
9	mir-6-2	no	no	63	mir-312	no	pharate
10	mir-6-3	no	no	64	mir-313	no	no
11	mir-7	pharate	pharate	65	mir-314	no	no
12	mir-8	no	no	66	mir-315	pharate	pharate
13	mir-9a	no	no	67	mir-316	no	no
14	mir-9b	embryonic	embryonic	68	mir-317	no	no
15	mir-9c	embryonic	embryonic	69	mir-318	no	no
16	mir-10	no	no	70	mir-375	no	no
17	mir-11	no	no	71	mir-927	pharate	pharate
18	mir-12	no	no	72	mir-929	no	no
19	mir-13b-1	no	no	73	mir-954	no	no
20	mir-13b-2	no	no	74	mir-955	no	no
21	mir-14	no	no	75	mir-956	no	no
22	mir-31a	no	no	76	mir-957	no	no
23	mir-33	no	no	77	mir-958	no	no
24	mir-34	no	no	78	mir-959	no	no
25	mir-79	no	no	79	mir-960	no	no
26	mir-87	no	no	80	mir-961	no	no
27	mir-92a	larval	larval	81	mir-962	no	no
28	mir-100	no	no	82	mir-963	no	no
29	mir-124	no	no	83	mir-964	no	no
30	mir-125	no	no	84	mir-965	pharate	pharate
31	mir-133	larval	larval	85	mir-966	no	no
32	mir-137	pharate	pharate	86	mir-967	no	no
33	mir-184	larval	pupal	87	mir-969	no	no
34	mir-190	pharate	pharate	88	mir-970	no	no
35	mir-193	no	no	89	mir-971	pharate	pharate
36	mir-219	pupal	pupal	90	mir-972	no	no
37	mir-252	no	no	91	mir-973	no	no
38	mir-263a	pupal	pupal	92	mir-974	no	no
39	mir-263b	no	no	93	mir-975	no	no
40	mir-274	no	no	94	mir-976	no	no
41	mir-275	no	no	95	mir-977	embryonic	embryonic
42	mir-276a	pharate	pharate	96	mir-978	no	no
43	mir-276b	no	no	97	mir-980	no	no
44	mir-277	no	no	98	mir-981	larval	larval
45	mir-278	no	no	99	mir-982	no	no
46	mir-279	pupal	pupal	100	mir-983-1	no	no
47	mir-280	no	no	101	mir-983-2	no	no
48	mir-281-1	no	no	102	mir-984	no	no
49	mir-282	no	no	103	mir-985	no	no
50	mir-284	no	embryonic	104	mir-986	no	no
51	mir-285	no	no	105	mir-987	no	no
52	mir-288	no	no	106	mir-988	no	no
53	mir-289	no	no	107	mir-989	no	no
54	mir-303	no	no	108	mir-990	no	no

	UAS-*mirRNA*	Lethality with *DMef2*-GAL4	Lethality with UAS*Dcr-2;* D*Mef2*-GAL4		UAS-*mirRNA*	Lethality with *DMef2*-GAL4	Lethality with UAS*Dcr-2;* D*Mef2*-GAL4
109	mir-991	no	no	162	mir-4976	no	no
110	mir-992	no	no	163	mir-iab-4	no	no
111	mir-993	no	no	164	mir-iab-4as	no	no
112	mir-994	no	no	165	mirror	no	no
113	mir-995	no	no				
114	mir-996	no	no				
115	mir-997	no	no				
116	mir-998	no	no				
117	mir-999	no	no				
118	mir-1000	no	no				
119	mir-1001	no	no				
120	mir-1002	no	no				
121	mir-1003	no	no				
122	mir-1004	no	no				
123	mir-1005	no	no				
124	mir-1006	no	no				
125	mir-1007	no	no				
126	mir-1008	no	no				
127	mir-1009	no	no				
128	mir-1010	no	no				
129	mir-1012	no	no				
130	mir-1013	no	no				
131	mir-1014	no	no				
132	mir-1015	no	no				
133	mir-1016	no	no				
134	mir-1017	no	no				
135	mir-2279	no	no				
136	mir-2280	no	no				
137	mir-2281	no	no				
138	mir-2282	no	no				
139	mir-2283	no	no				
140	mir-2489	no	no				
141	mir-2490	no	no				
142	mir-2491	no	no				
143	mir-2492	no	no				
144	mir-2493	no	no				
145	mir-2494	no	no				
146	mir-2495	no	no				
147	mir-2496	no	no				
148	mir-2497	no	no				
149	mir-2498	no	no				
150	mir-2499	no	no				
151	mir-2500	no	no				
152	mir-2501	no	no				
153	mir-2794	no	no				
154	mir-3642	no	no				
155	mir-3643	no	no				
156	mir-3644	no	no				
157	mir-3645	no	no				
158	mir-4951	no	no				
159	mir-4966	no	no				
160	mir-4968	no	no				
161	mir-4969	no	no				
